# Flutamide Induces Hepatic Cell Death and Mitochondrial Dysfunction via Inhibition of Nrf2-Mediated Heme Oxygenase-1

**DOI:** 10.1155/2018/8017073

**Published:** 2018-07-02

**Authors:** Li Zhang, Jiabin Guo, Qiang Zhang, Wei Zhou, Jin Li, Jian Yin, Lan Cui, Tingfen Zhang, Jun Zhao, Paul L. Carmichael, Alistair Middleton, Shuangqing Peng

**Affiliations:** ^1^Academy of Military Medical Sciences, Beijing 100850, China; ^2^Evaluation and Research Centre for Toxicology, Institute of Disease Control and Prevention, PLA, Beijing 100071, China; ^3^Department of Environmental Health, Rollins School of Public Health, Emory University, Atlanta, GA 30322, USA; ^4^Unilever Safety and Environmental Assurance Center, Colworth Science Park, Sharnbrook, Bedfordshire MK44 1LQ, UK

## Abstract

Flutamide is a widely used nonsteroidal antiandrogen for prostate cancer therapy, but its clinical application is restricted by the concurrent liver injury. Increasing evidence suggests that flutamide-induced liver injury is associated with oxidative stress, though the precise mechanism is poorly understood. Nuclear factor erythroid 2-related factor 2 (Nrf2) is a master transcription factor regulating endogenous antioxidants including heme oxygenase-1 (HO-1). This study was designed to delineate the role of Nrf2/HO-1 in flutamide-induced hepatic cell injury. Our results showed that flutamide concentration dependently induced cytotoxicity, hydrogen peroxide accumulation, and mitochondrial dysfunction as indicated by mitochondrial membrane potential loss and ATP depletion. The protein expression of Nrf2 and HO-1 was induced by flutamide at 12.5 *μ*M but was downregulated by higher concentrations of flutamide. Silencing either Nrf2 or HO-1 was found to aggravate flutamide-induced hydrogen peroxide accumulation and mitochondrial dysfunction as well as inhibition of the Nrf2 pathway. Moreover, preinduction of HO-1 by Copp significantly attenuated flutamide-induced oxidative stress and mitochondrial dysfunction, while inhibition of HO-1 by Snpp aggravated these deleterious effects. These findings suggest that flutamide-induced hepatic cell death and mitochondrial dysfunction is assoicated with inhibition of Nrf2-mediated HO-1. Pharmacologic intervention of Nrf2/HO-1 may provide a promising therapeutic approach in flutamide-induced liver injury.

## 1. Introduction

Flutamide (Flu, 2-methyl-N-[4-nitro-3-(trifluoromethyl) phenyl] propanamide) is an oral, widely used nonsteroidal antiandrogen approved for the treatment of prostate cancer [1]. Although generally considered safe, flutamide therapy is compromised by the occurrence of hepatotoxicity [2]. Flutamide can induce cholestasis, jaundice, and liver necrosis, which may eventually require a liver transplant. Because of the concurrent hepatotoxicity, flutamide received a black box warning label by the FDA in 1999 [3]. Mitochondrial injury is increasingly proposed as a putative hazard of flutamide though the susceptibility factors which are unknown [4]. Experimental evidence has shown that flutamide can inhibit mitochondrial complex I [5], induce ATP depletion [6], and disrupt mitochondrial membrane potential (MMP) [7] in cultured hepatic cells. Studies using different mouse models have revealed that flutamide impaired glucose homeostasis and triggered mitochondrial dysfunction [8]. These findings suggest that mitochondria play a critical role in flutamide-induced liver injury. However, the mechanisms of flutamide-induced mitochondrial damage and liver injury remain elusive.

Mitochondria are the main sites of oxidative phosphorylation and energy metabolism. Meanwhile, mitochondria are also major intracellular sources of reactive oxygen species (ROS). Mitochondrial ROS can physiologically act as important redox signaling molecules and play an important role in maintaining cellular oxidant/antioxidant balance. However, excessive ROS production can cause mitochondrial damage that may eventually lead to liver diseases [9]. Emerging evidence implicates that flutamide-induced hepatotoxicity is associated with ROS-mediated oxidative stress [10]. During the metabolism of flutamide in the liver, it is oxidatively metabolized by microsomal metabolic enzymes and turned into electrophilic metabolites. Meanwhile, ROS are also produced as by-products. It has been shown that flutamide induces ROS formation and other prooxidant radicals leading to oxidative stress and mitochondrial dysfunction in primary cultured hepatocytes [4].

Nuclear factor-erythroid 2-related factor 2 (Nrf2) is a master transcription factor regulating the oxidative stress response [11]. Nrf2 is particularly important during times of severe oxidative stress through its ability to regulate the expression of antioxidant proteins and phase II enzymes, such as heme oxygenase-1 (HO-1), NAD(P)H: quinone oxidoreductase 1 (NQO1), and glutathione-S-transferases (GST). Recent studies have shown that HO-1 plays a key role in counteracting oxidative stress and mitochondrial damage [12, 13]. HO-1 is a highly inducible antioxidant enzyme that can be induced under a number of conditions, such as oxidative stress, infection, inflammation, and hypoxia [14]. HO-1 converts potentially toxic heme released by mitochondria into the antioxidant biliverdin. Degradation of heme results in the release of iron and production of CO, which functions as a key signal that independently upregulates cellular iron-responsive and antioxidant defense [14]. In addition, CO can bind to the reduced A3 heme of cytochrome C oxidase (COX), which enhances mitochondrial hydrogen peroxide release and contributes to retrograde activation of mitochondrial biogenesis.

Increasing evidence suggests that the Nrf2 pathway is critically involved in drug-induced liver injury [15]. It is implicated that many drug-induced hepatotoxicity is attributed to the inhibition of the Nrf2 pathway while its activation was found to provide effective protection against liver injury [16–18]. However, the precise role of HO-1 in flutamide-induced hepatotoxicity is poorly understood. According to the TT21C report entitled “Toxicity Testing in the Twenty First Century: Vision and Strategy” released by the USA National Research Council (NRC, 2007), toxicity pathway-based drug safety assessments have been proposed as a central part in toxicity testing in the 21st century. As depicted by that report which was regarded as a milestone in toxicology, toxicity testing strategy is undergoing a tremendous transformation from traditional animal tests to in vitro approaches and other nonanimal alternatives that primarily and preferably use human-originated cells/cell lines. In compliance with this report, we utilized HepG2 cells to explore the role of the Nrf2/HO-1 pathway in flutamide-induced toxicity. We demonstrated that flutamide-induced hepatic cell death, oxidative stress, and mitochondrial dysfunction are through the inhibition of Nrf2-mediated HO-1 induction. Preinduction of HO-1 protected against flutamide-induced hepatic mitochondrial dysfunction. In contrast, inhibition of HO-1 exacerbated flutamide-induced hepatotoxicity. These findings highlight an important role of HO-1 in flutamide-induced liver injury and suggest that Nrf2/HO-1 may be a promising therapeutic target for preventing and treating drug-induced liver injury.

## 2. Materials and Methods

### 2.1. Cell Culture and Drug Treatments

HepG2 cells were obtained from Shanghai Cell Line Bank (Shanghai, China) and routinely cultured in MEM supplemented with 10% FBS, 100 U/mL penicillin, and 100 *μ*g/mL streptomycin. Cell cultures were grown at 37°C in humidified incubators containing an atmosphere of 5% CO_2_. All cells used in our experiments were up to passage 20. Cells were treated with flutamide (Sigma-Aldrich, USA) for 24 h at various concentrations as indicated. In some experiments, cells were pretreated with or without 20 *μ*M tin protoporphyrin (Snpp, Sigma-Aldrich) or 10 *μ*M cobalt protoporphyrin (Copp, Sigma-Aldrich) for 1 h followed by treatment with 50 *μ*M flutamide.

### 2.2. Evaluation of Cytotoxicity

Cytotoxicity was evaluated by the determination of cell viability and lactate dehydrogenase (LDH) leakage. Cell viability was assessed by using a commercial cell counting Kit-8 (CCK8, Dojindo Molecular Technologies, Japan) according to the manufacturer's instruction. Briefly, immediately after drug treatment, cells were incubated with CCK-8 working solution at 37°C for 2 h. Subsequently, the absorbance at 450 nm was monitored using a microplate reader (Multiskan MK3, Thermo Fisher Scientific, USA). Data were normalized to the values from control cultures without drug treatment which were considered 100% survival.

LDH release into the medium was measured to estimate the extent of cell damage. At the end of drug treatment, supernatants of culture media were collected for LDH assay. The activity of LDH was measured by recording the absorbance at 490 nm using a cytotoxicity LDH assay kit (Beyotime Biotechnology, China).

### 2.3. Determination of Hydrogen Peroxide Content

Hydrogen peroxide was measured using a hydrogen peroxide assay kit based on ferrous oxidation of xylenol orange assay according to the manufacturer's instructions (Beyotime Institute of Biotechnology, China). Cells were freshly collected and lysed immediately after drug treatments. Cell lysate was centrifuged at 12,000*g* at 4°C for 5 min. The supernatant was collected and incubated with a hydrogen peroxide detecting reagent at room temperature for 30 min. Absorbance at 560 nm was then monitored by a microplate reader.

### 2.4. Detection of Mitochondrial Membrane Potential

Mitochondrial membrane potential was indicated by a MitoTracker® probe (Invitrogen) which contains a mildly thiol-reactive chloromethyl moiety for labeling mitochondria. After completion of drug treatment, cells were incubated with staining solution containing 100 nM MitoTracker probe in the dark at 37°C for 30 min. Thereafter, cells were washed at least thrice with prewarmed PBS to completely remove extra probe. The fluorescence intensity of MitoTracker probe was measured using a FACS Calibur flow cytometer (Becton Dickinson, USA).

### 2.5. Determination of ATP

Cellular ATP content was determined by ATP colorimetric assay (BioVision) by utilizing the phosphorylation of glycerol to generate a product that is quantified by colorimetric methods. Samples were collected and processed according to the manufacturer's instruction. In brief, cells were lysed in an ATP assay buffer, followed by deproteinizing using a deproteinization sample preparation kit (BioVision). The samples were then mixed with ATP assay buffer, along with reaction mix and an ATP probe. The reaction system was incubated in the dark at room temperature for 30 min. Thereafter, absorbance at 570 nm was monitored by a microplate reader.

### 2.6. siRNA Transfection

Nrf2 knockdown cell model and HO-1 knockdown cell model were established by transfection with specific siRNA as we previously reported [19]. In brief, cells were plated in 6-well plates and transiently transfected with 70 nM of small interfering oligonucleotide (siRNA) against Nrf2 or HO-1 (Santa Cruz Biotechnology, USA) or control nonspecific oligonucleotide (ConsiRNA) using lipid-based transfection system (Lipofectamine 3000, Thermo Fisher Scientific) for 5 h. Thereafter, cells were allowed to recover in fresh media for 24 h according to the manufacturer's protocol. The efficiency of Nrf2 or HO-1 knockdown was confirmed by the detection of the mRNA and protein level quantified by qPCR and Western blot, respectively.

### 2.7. Western Blotting Analysis

Cells were lysed with ice-cold RIPA buffer (Applygen Technologies) containing protease and phosphatase inhibitors (Applygen Technologies). Protein samples were collected and resolved by 8% or 12% SDS-PAGE and were then transferred to polyvinylidene difluoride membranes (PVDF) (Millipore, USA). Membranes were blocked with 5% nonfat milk in TBS containing 0.1% Tween 20 (TBS-T) for 4 h and incubated with primary antibodies at 4°C overnight, followed by 1 h incubation with horseradish peroxidase-conjugated secondary antibodies (Santa Cruz Biotechnology) at room temperature. The blots were detected using ECL detection system (Applygen Technologies) and recorded by chemiluminescence imaging analysis. Images were analyzed using ImageJ software (National Institutes of Health, USA).

### 2.8. Statistical Analysis

All values were expressed as the mean ± SD from 3 independent experiments. Statistical analyses were performed by one-way ANOVA followed by Dunnett's test. Data were analyzed and presented with PASW Statistics 18.0 software (SPSS Inc., USA). A *P* value < 0.05 was considered statistically significant.

## 3. Results

### 3.1. Characterization of the Cytotoxicity of Flutamide in HepG2 Cells

**Figure 1:**
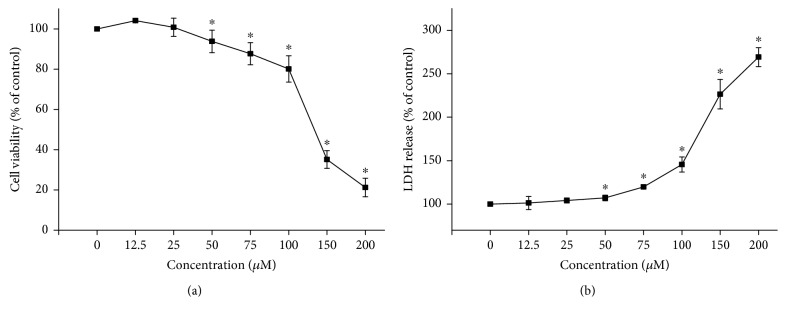
Flutamide concentration dependently induced cytotoxicity in HepG2 cells. Cells were treated with flutamide at various concentrations (0–200 *μ*M) for 24 h. Cytotoxicity was evaluated by CCK-8 assays for cell viability (a) and LDH assays for LDH leakage (b). Data are presented as the mean ± SD from 3 independent experiments. ^∗^*P* < 0.05 compared with the cells in the control group without drug treatment.

The cytotoxicity of flutamide in HepG2 cells was evaluated by cell viability and LDH leakage. Cells were exposed to flutamide for 24 h at various concentrations ranging from 0 to 200 *μ*M. Compared to cells in the control group, cells treated with flutamide at concentrations higher than 25 *μ*M showed significant and concentration-dependent decreases in cell viability with an LC_50_ (lethal concentration required to cause 50% reduction in cell viability) of 133 *μ*M (Figure 1(a)). The activity of LDH in media was also concentration dependently increased by flutamide. In line with cell viability assay, a significant increase in LDH activity was found in cells treated with flutamide at concentrations higher than 25 *μ*M (Figure 1(b)).

### 3.2. Flutamide-Induced ROS Accumulation and Mitochondrial Dysfunction

**Figure 2:**
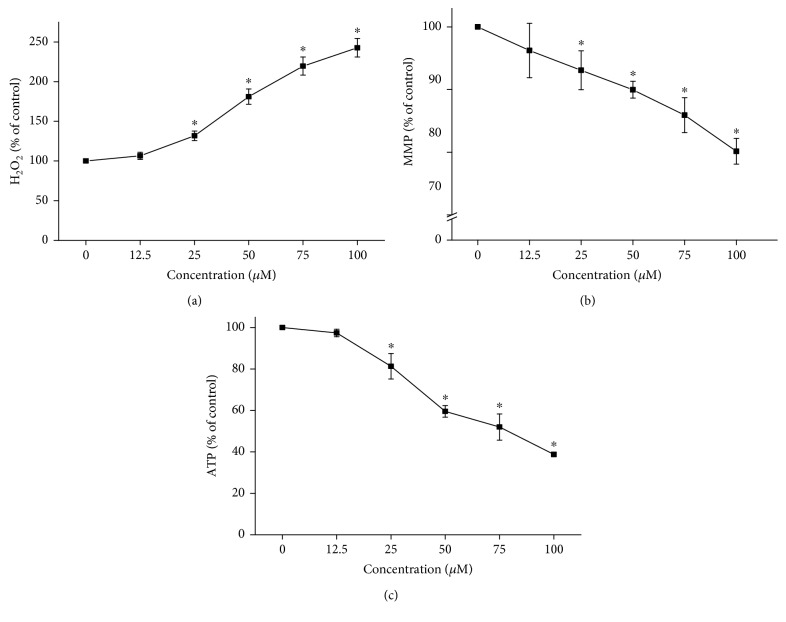
Flutamide increased hydrogen peroxide (H_2_O_2_) accumulation and elicited mitochondrial dysfunction by a concentration-dependent manner. Cells were treated with flutamide at the concentrations up to 100 *μ*M for 24 h. Hydrogen peroxide content was analyzed by a commercial kit based on ferrous oxidation of xylenol orange assay (a). Mitochondrial dysfunction was indicated by determination of mitochondrial membrane potential (b) and ATP content. Data are presented as the mean ± SD from 3 independent experiments. ^∗^*P* < 0.05 compared with the cells in the control group without drug treatment.

Excessive ROS production has been implicated as an important causative factor for flutamide-induced hepatotoxicity [20]. Hydrogen peroxide is one of the main types of ROS that can directly attack cellular component, such as lipid, protein, and DNA, leading to oxidative damage [21]. As shown in Figure 2(a), flutamide increased hydrogen peroxide levels by a concentration-dependent manner. Compared with cells in the control group, hydrogen peroxide contents were significantly increased in cells treated with flutamide at concentrations higher than 12.5 *μ*M. For cells treated with flutamide at 100 *μ*M, the hydrogen peroxide content was increased by 2.4-fold compared with cells in the control group.

Mitochondrial function was evaluated by the determination of mitochondrial membrane potential and ATP production. As shown in Figure 2(b), flutamide was found to concentration dependently decrease mitochondrial membrane potential. Significant mitochondrial membrane potential loss was found in the cells treated with flutamide at a concentration over 12.5 *μ*M compared to cells in the control group. Similar results were found in ATP assays. As shown in Figure 2(c), 28.7%, 40.5%, 48.0%, and 51.2% reductions in ATP levels were found, respectively, for cells treated with flutamide at 25, 50, 75, and 100 *μ*M compared to cells in the control group.

### 3.3. Flutamide-Perturbed Nrf2/HO-1 Pathway

**Figure 3:**
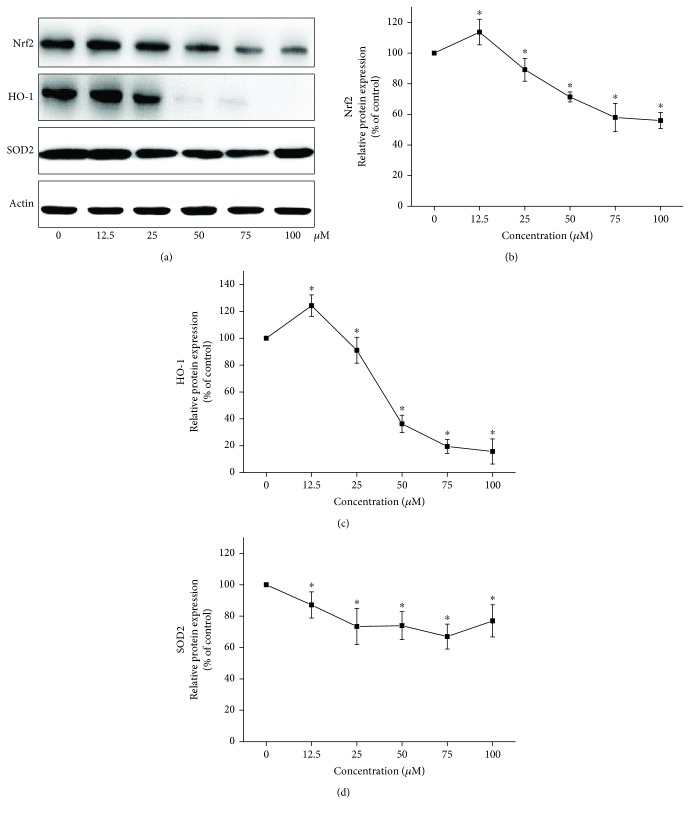
Effects of flutamide on the Nrf2/HO-1 pathway. Cells were treated with flutamide at the concentrations ranging from 12.5 *μ*M to 100 *μ*M for 24 h. The protein expressions of Nrf2, HO-1, and SOD2 were measured by Western blot. Representative images of blots are shown in (a). The protein expressions of Nrf2 (b), HO-1 (c), and SOD2 (d) were quantitatively analyzed by ImageJ software. Data are presented as the mean ± SD from 3 independent experiments. ^∗^*P* < 0.05 compared with the cells in the control group without drug treatment.

To investigate the effect of flutamide on the Nrf2/HO-1 pathway, the protein expressions of Nrf2, HO-1, and superoxide dismutase-2 (SOD2) were analyzed with Western blot (Figure 3(a)). Protein levels of Nrf2 (Figure 3(b)) and HO-1 (Figure 3(c)) were slightly increased by flutamide at 12.5 *μ*M but were significantly decreased by higher concentrations of flutamide. Compared to the cells in the control group, the protein levels of Nrf2 were increased by 13.7% and decreased by 11.0%, 28.7%, 42.1, and 44.0% for cells treated with flutamide at 12.5, 25, 50, 75, and 100 *μ*M, respectively. Remarkable changes were also found in HO-1 protein expression, which was increased by 24.3% but decreased by 9.0%, 63.7%, 80.6%, and 84.3%, respectively, for cells treated with flutamide at 12.5, 25, 50, 75, and 100 *μ*M, respectively (Figure 3(d)). In contrast, the protein expression of SOD2 was inhibited by flutamide at all concentrations tested with decreases ranging from 12.9% to 33.1%.

### 3.4. Knockdown of Nrf2/HO-1 Aggravated Flutamide-Induced Oxidative Stress, Mitochondrial Dysfunction, and Inhibition of Nrf2/HO-1 Pathway

To evaluate the role of the Nrf2/HO-1 pathway in flutamide-induced hepatotoxicity, Nrf2 knockdown and HO-1 knockdown cell models were established. HepG2 cells were treated with Nrf2 or HO-1 siRNA at a concentration at which no obvious cytotoxicity was elicited. The efficiency of Nrf2 and HO-1 knockdown was confirmed by RT-PCR and Western blot to detect mRNA and protein levels, respectively. The efficiency of Nrf2 and HO-1 knockdown efficiency at protein level were approximately 50% and 40% (data not shown).

**Figure 4:**
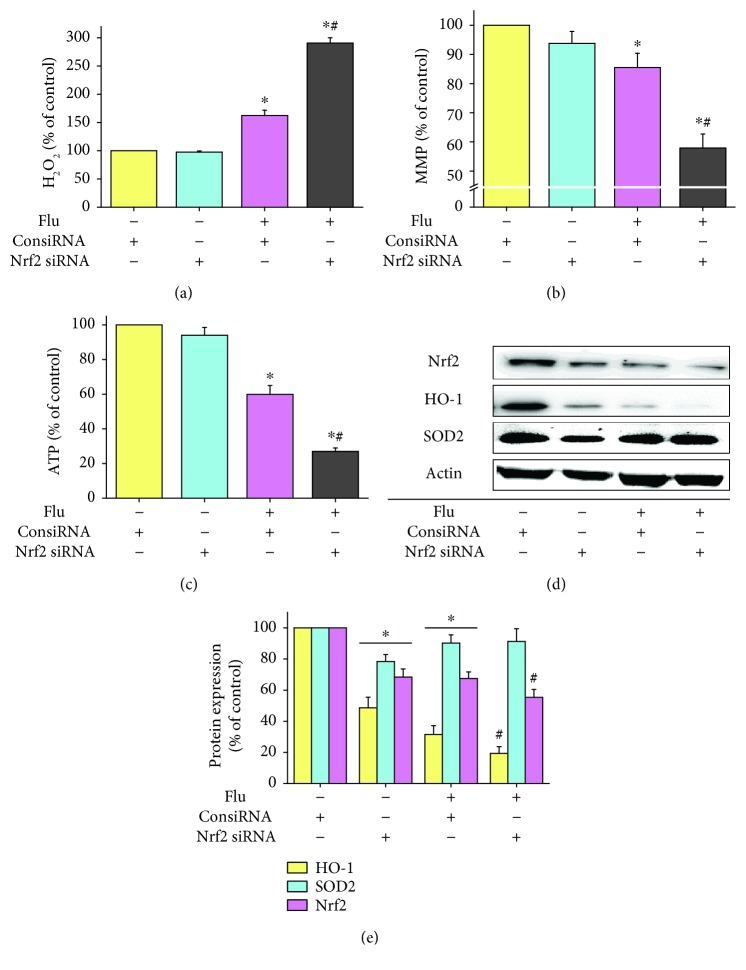
Knockdown of Nrf2 aggravated flutamide-induced oxidative stress, mitochondrial dysfunction, and inhibition of the Nrf2/HO-1 pathway. ConsiRNA-treated cells and Nrf2-silenced cells were exposed to flutamide at 50 *μ*M for 24 h. Afterward, the levels of hydrogen peroxide (a), mitochondrial membrane potential (b), and ATP (c) were determined. The protein expressions of Nrf2, HO-1, and SOD2 were analyzed by Western blot (d) and quantified by ImageJ (e). Data are presented as the mean ± SD from 3 independent experiments. ^∗^*P* < 0.05 compared with ConsiRNA-treated cells without drug treatment; ^#^*P* < 0.05 compared with ConsiRNA-treated cells with flutamide treatment.

Nrf2 knockdown by itself showed no tangible effect on the hydrogen peroxide level, mitochondrial membrane potential, and ATP content. However, compared to cells treated with ConsiRNA, 50 *μ*M flutamide-induced hydrogen peroxide accumulation, mitochondrial membrane potential loss, and ATP depletion were significantly aggravated in Nrf2 knockdown cells. Flutamide elevated the levels of hydrogen peroxide by 1.7- and 2.9-fold, respectively, for ConsiRNA-treated cells and Nrf2 knockdown cells (Figure 4(a)). The mitochondrial membrane potential was decreased by 14.5% and 42.1%, respectively, for ConsiRNA-treated cells and Nrf2 knockdown cells following flutamide exposure (Figure 4(b)). Similarly, flutamide was found to induce 34.5% and 73.0% reduction in ATP levels for ConsiRNA-treated cells and Nrf2 knockdown cells, respectively, (Figure 4(c)). As expected, the protein expressions of Nrf2, HO-1, and SOD2 were significantly decreased in Nrf2 knockdown cells (Figures 4(d) and 4(e)). Flutamide-induced inhibition of Nrf2 and HO-1 was further aggravated by Nrf2 knockdown. Interestingly, no significant difference was found in the protein expression of SOD2 between ConsiRNA-treated cells and Nrf2 knockdown cells following flutamide exposure.

**Figure 5:**
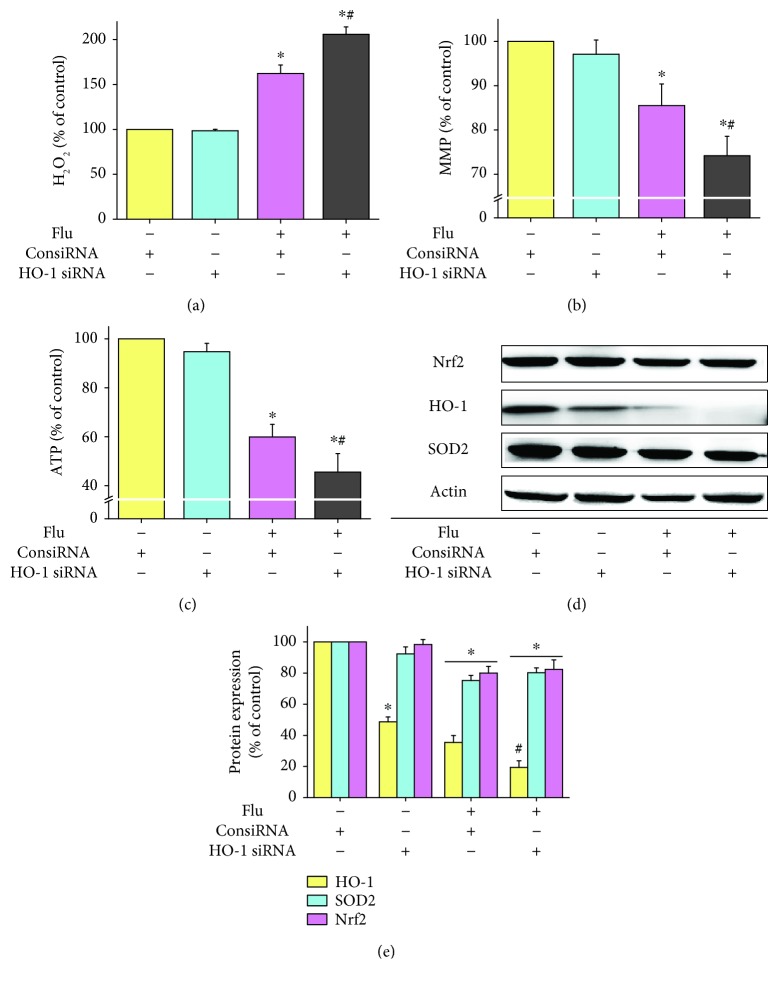
Knockdown of HO-1 exacerbated flutamide-induced oxidative stress and mitochondrial dysfunction. ConsiRNA-treated cells and Nrf2-silenced cells were exposed to flutamide at 50 *μ*M for 24 h. Afterward, the levels of hydrogen peroxide (a), mitochondrial membrane potential (b), and ATP (c) were determined. The protein expressions of Nrf2, HO-1, and SOD2 were analyzed by Western blot (d) and quantified by ImageJ (e). Data are presented as the mean ± SD from 3 independent experiments. ^∗^*P* < 0.05 compared with ConsiRNA-treated cells without drug treatment; ^#^*P* < 0.05 compared with ConsiRNA-treated cells with flutamide treatment.

Knockdown of HO-1 was also found to exacerbate flutamide-induced oxidative stress and mitochondrial function. The hydrogen peroxide level, mitochondrial membrane potential, and ATP level were not affected by HO-1 silencing (Figures 5(a)–5(c)). HO-1 knockdown only significantly decreased the expression of HO-1 but did not alter the protein expression of Nrf2 and SOD2 (Figures 5(d) and 5(e)). Compared to cells without drug treatment, the hydrogen peroxide level was increased by 2.1-fold while the mitochondrial membrane potential and ATP content were decreased by 25.9% and 54.5%, respectively, in HO-1 knockdown cells treated with 50 *μ*M. Silencing of HO-1 exacerbated flutamide-induced inhibition of HO-1 protein expression, but no significant difference was found in the protein expression of Nrf2 and SOD2 between ConsiRNA-treated cells and HO-1 knockdown cells following flutamide exposure.

### 3.5. Effects of HO-1 Interventions on Flutamide-Induced Oxidative Stress and Mitochondrial Dysfunction

**Figure 6:**
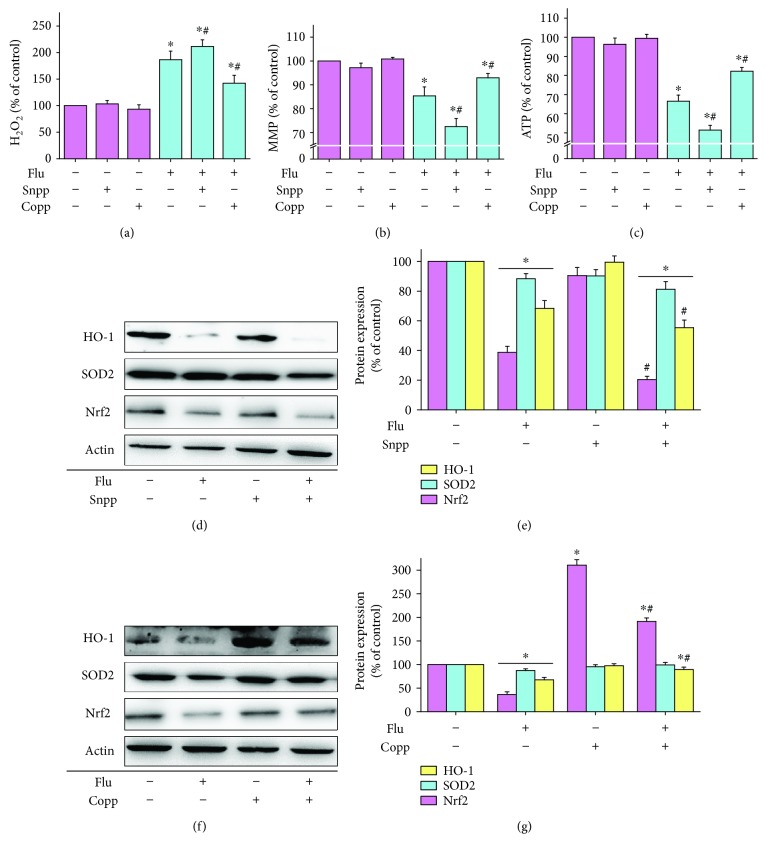
Effects of HO-1 interventions on flutamide-induced oxidative stress and mitochondrial dysfunction. Cells were pretreated with or without 20 *μ*M Snpp/10 *μ*M Copp for 1 h followed by treatment with 50 *μ*M flutamide. After completion of drug treatments, the levels of hydrogen peroxide (a), mitochondrial membrane potential (b), and ATP (c) as well as the protein expressions of Nrf2, HO-1, and SOD2 were analyzed (d–f). Data are presented as the mean ± SD from 3 independent experiments. ^∗^*P* < 0.05 compared with the cells in the control group without drug treatment; ^#^*P* < 0.05 compared with the cells treated with flutamide.

Given the prominent alterations of HO-1 following flutamide treatment, we further investigated the effect of HO-1 interventions on flutamide-mediated hepatic injury and Nrf2/HO-1 pathway perturbation. Cells were pretreated with 10 *μ*M Copp (HO-1 inducer) or 20 *μ*M Snpp (HO-1 inhibitor) for 1 h before flutamide (50 *μ*M) treatment. Copp significantly blocked flutamide-induced oxidative stress and mitochondrial dysfunction, while inhibition of HO-1 by Snpp remarkably aggravated these deleterious effects. Compared to cells in the control group without drug treatment, the levels of hydrogen peroxide in cells treated with flutamide, Snpp plus flutamide, and Copp plus flutamide were elevated by 1.9-, 2.1-, and 1.4-fold, respectively (Figures 6(a)–6(c)). The mitochondrial membrane potential and ATP content were reduced by 27.5 and 48.6% in cells treated with Snpp plus flutamide. In comparison, cells treated with Copp only showed a 7.0% reduction in mitochondrial membrane potential and 17.8% decrease in ATP content. Results from Western blot analysis showed that Snpp pretreatment significantly potentiated flutamide-induced inhibition of Nrf2 and HO-1 protein expression (Figures 6(d) and 6(e)), while Copp can ameliorate these changes by flutamide (Figures 6(f) and 6(g)). Neither Snpp nor Copp has an obvious effect on flutamide-induced inhibition of SOD2 expression.

## 4. Discussion

Flutamide is an antiandrogen drug that is widely used for the treatment of prostate cancer. However, the therapeutic effects of flutamide have been overshadowed by reports of liver dysfunction in 1–10% of its users [2, 22]. This flutamide-induced liver injury is not acute but delayed [3, 23]. In most cases, flutamide caused liver dysfu1nction after a latency period of approximately 16 weeks [24]. Our results demonstrated that 24 h treatment of HepG2 cells with flutamide concentration dependently induced reduction of cell viability and LDH leakage. Flutamide did not elicit significant effects on these cytotoxicity indices at the concentration of 12.5 and 25 *μ*M. Thus, the observed adverse effect level (NOAEL) of flutamide-induced cytotoxicity in HepG2 cells is 25 *μ*M. Flutamide-induced mitochondrial damage was evaluated by the determination of mitochondrial membrane potential and ATP production. Our results showed that flutamide concentration dependently decreased mitochondrial membrane potential and reduced the APT level. Significant mitochondrial toxic effects were found at the concentration of 25 *μ*M at which no significant cytotoxicity was observed induced, suggesting mitochondria as a sensitive target of flutamide-induced hepatocyte toxicity.

Mitochondria are thought to play a critical role in the development and pathogenesis of drug-induced liver injury, not only due to their role as the main source of endogenous ROS but also due to the role as the target of ROS attack. It has been shown that the accumulation of ROS in hepatic cells is an essential step in flutamide hepatotoxicity [4]. It is implicated that flutamide can promote ROS generation, especially in the mitochondria, by multiple mechanisms [10]. For instance, flutamide has been shown to inhibit mitochondrial complex I leading to superoxide production through the reduction of molecular oxygen by the NADH/ubiquinone oxidoreductase [25]. Flutamide can also produce many reactive oxidants during its metabolism in the liver. In the present study, flutamide was found to increase the level of hydrogen peroxide in a concentration-dependent manner; however, the specific source of the observed flutamide-elicited ROS accumulation still needs further investigation.

Excessive ROS generation can disrupt mitochondrial membrane potential, induce mitochondrial dysfunction, and compromise the capacity of the antioxidant defense system [26]. The mitochondria have been indicated as important targets of flutamide-mediated adverse effects in the liver. It has been shown that flutamide can alter mitochondrial morphology and profoundly downregulate genes associated with fatty acid *β*-oxidation and upregulate genes related to antioxidant defense in hepatocytes [6]. The loss of ATP was also found to be a critical event in the cytotoxicity of flutamide caused by its ability to target complex I of the electron transport chain and impair oxidative phosphorylation [6]. Consistent with the observation of increased hydrogen peroxide accumulation, our results demonstrated that flutamide induced mitochondrial membrane potential loss and reduction of ATP.

Nrf2 is ubiquitously expressed throughout human tissues, with high expression in detoxification organs, especially the liver. Nrf2 serves as a major regulator of the cellular antioxidant defense pathway by which hepatic cells combat oxidative stress [27, 28]. During oxidative stress, Nrf2 activation is initiated to transcriptionally regulate its target genes to detoxify and eliminate potentially harmful exogenous chemicals and their metabolites [29, 30]. Growing evidence has suggested that the activation of the Nrf2 pathway is a key mechanism underlying the protective effects of many pharmacological agents [31, 32]. In contrast, the inhibition of the Nrf2 pathway has been implicated as a critical cause leading to the adverse effects of several chemicals [33]. Nrf2 knockout mice and Nrf2-deficient cells have been found to be more sensitive to oxidative injury [34–36]. In the present study, the protein expression of Nrf2 was slightly increased by flutamide at a lower concentration (12.5 *μ*M) but was significantly inhibited by higher concentrations (≥50 *μ*M). Knockdown of Nrf2 was found to significantly sensitize the cells to flutamide-induced ROS accumulation and mitochondrial dysfunction. The changes of Nrf2 were mirrored by HO-1 and SOD2, though the magnitude of SOD2 alternation was less than Nrf2 and HO-1. These findings suggest that the Nrf2 pathway was activated by flutamide at a low dose to counteract oxidative stress. However, cells exposed to flutamide at a higher dose might undergo saturated Nrf2 pathway activation, thus failing to combat increased ROS generation, consequently leading to inhibition of this pathway and mitochondrial dysfunction.

**Figure 7:**
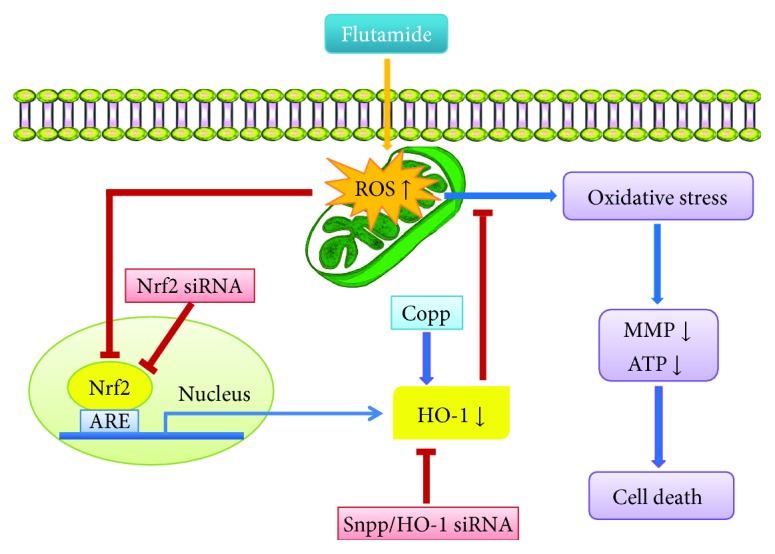
Schematic representation of the role of Nrf2-mediated HO-1 in flutamide-induced hepatic cell death and mitochondrial dysfunction.

HO-1 is implicated as one of the most important downstream antioxidant targets of Nrf2 protecting against oxidative stress damage [37–39]. Increasing evidence suggests that HO-1 is particularly important in regulating mitochondrial function and energy metabolism as well as oxidative stress [40, 41]. HO-1 represents a prime antioxidant defense mechanism against mitochondrial oxidative stress through several ways [42–44]. The induction of HO-1 leads to increased cellular CO production, which generates a redox signal for the induction of mitochondrial biogenesis [45]. HO-1 has also been implicated in the regulation of mitophagy and mitochondrial quality control cycle [46]. Our results demonstrated that HO-1 was induced by flutamide at a low concentration but was inhibited by high concentrations. Compared to the changes of Nrf2 and SOD2, the alteration of HO-1 was much more prominent, indicating HO-1 as a sensitive marker of liver injury following flutamide treatment. A recent study by Teppner et al. showed that the gene expression of HO-1 was increased but no obvious cytotoxic effects were induced in *in vitro* cultured rat hepatocytes treated with flutamide at 50 and 100 *μ*M, which are similar to the concentrations used in the present study [47]. This discrepancy with our results might involve several causes such as different cell models and experimental parameters. In addition, HO-1 silence significantly augmented flutamide-induced oxidative stress and mitochondrial dysfunction. Preinduction of HO-1 by Copp significantly attenuated flutamide-induced ROS accumulation and mitochondrial dysfunction, while inhibition of HO-1 by Snpp remarkably aggravated the deleterious effects. Taken together, these findings suggested that HO-1 was particularly important in flutamide-induced hepatotoxicity (Figure 7).

In conclusion, our results suggested that flutamide-induced hepatotoxicity involves, at least in part, the inhibition of the Nrf2/HO-1 pathway and highlighted an important role of HO-1 in flutamide-induced hepatic mitochondrial injury. The induction of HO-1 prevented flutamide-induced hepatic mitochondrial dysfunction while inhibition of HO-1 exacerbated the adverse effects. These findings provide new evidence to understand the mechanism of flutamide-induced liver injury. Given that oxidative stress and mitochondrial dysfunction underlie the liver injury induced by various drugs, the results obtained from the present study suggest the Nrf2 pathway, particularly HO-1, as a potential therapeutic target for patients with drug-induced adverse effects in the liver.

## Data Availability

The authors are willing to share the detailed/raw data in private with interested researchers.

## Additional Points

*Highlights.* Flutamide induces cell death, oxidative stress, and mitochondrial dysfunction. The Nrf2/HO-1 pathway was perturbed by flutamide. Knockdown of Nrf2/HO-1 aggravates flutamide-induced hepatic cell injury. Induction of HO-1 prevents hepatic oxidative stress and mitochondrial dysfunction. Inhibition of HO-1 exacerbates flutamide-induced hepatotoxicity.
